# Experimental and Numerical Characterization of Rigid Polyurethane Foam for Kinetic Collision Absorption Systems—Ogden Material Model

**DOI:** 10.3390/polym18141729

**Published:** 2026-07-14

**Authors:** Francis Franklin, Will Nightingale, Jovan Tanasković, Zorana Golubović

**Affiliations:** 1 School of Engineering, Newcastle University, Newcastle upon Tyne NE1 7RU, UK; francis.franklin@newcastle.ac.uk (F.F.);; 2 Faculty of Mechanical Engineering, University of Belgrade, 11120 Belgrade, Serbia; zzgolubovic@mas.bg.ac.rs

**Keywords:** energy absorption, rigid PU foam, numerical model, foam properties, absorption power, experimental research, Ogden foam model

## Abstract

Rigid polyurethane foam was evaluated as a filler material for a tubular railway vehicle energy absorber. Cubic samples cut from a cylindrical PU foam sample with a density of 175 kg/m^3^ were tested under quasi-static uniaxial compression to determine the material’s compressive response and provide input data for finite element modelling. The experimental results showed a non-linear stress–strain response typical of cellular foams, while samples from the central region of the cylinder exhibited a lower stress response than those from the outer region. An Ogden foam material model was calibrated in Ansys using compression data obtained by experimental tests and then applied to numerical models of three absorber configurations: an empty steel tube, a fully foam-filled steel tube, and a foam-filled tube with an additional concentric steel core. The simulations compared the force–stroke response and absorbed energy of each configuration under quasi-static axial loading through a conical bushing. Over a 60 mm stroke, compared to the empty tube, the fully foam-filled tube absorbed an additional 16% energy and the concentric-core configuration absorbed an additional 9.5%. These results indicate that rigid PU foam filling can improve the quasi-static energy absorption capacity of tubular railway collision absorbers.

## 1. Introduction

As the speed at which trains can operate increases, the need for suitable safety systems on railcars becomes paramount in order to guarantee the protection of the people and goods on board [1]. Polymeric foams are commonly used for their compressive capabilities as a means of protection against high-speed impacts [2]. Rigid polyurethane (PU) foam is a type of polymer foam and is relatively inexpensive and lightweight as well as capable of high energy absorption whilst maintaining a relatively low stress level [3]. PU foam is compressible, meaning its volume can change when pressure is applied to it. The material’s ability to recover from elastic deformations is due to the crosslinking polymer chains. When a load is applied to the foam, it will compress and convert the kinetic energy to potential energy, heat, and friction. These factors make it highly suitable to be used as a mechanical-energy-absorbing material under compressive loading in railway vehicles to reduce the magnitude of forces subject to the vehicle body in the event of a high-speed collision [4]. This, therefore, leads to a great interest in characterizing the mechanical properties of such a material.

**Figure 1:**
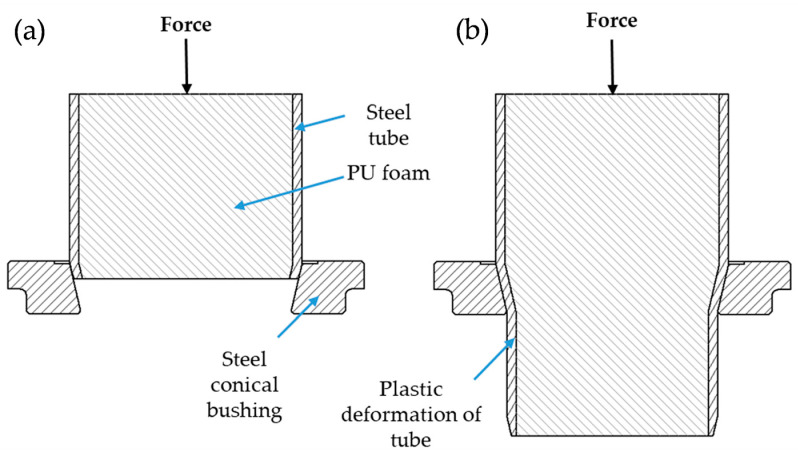
Collision absorber diagram (**a**) at start of force application, and (**b**) post force application.

An energy absorber in a vehicle has the primary role of reducing the risk of personal injury to the people on board and damage of goods in the event of a collision by absorbing impact energy and limiting the amount of distortion of the vehicle body [5]. Modern railway crash-energy management systems commonly combine several passive safety components. At lower collision speeds, coupler–buffer systems are typically the primary energy-absorbing elements, while, at higher collision speeds, the crash energy is increasingly dissipated by deformation tubes, crush boxes, deformable anti-climbers, and vehicle-end structures. Previous railway crashworthiness studies and reviews have emphasized that these components are used to control progressive deformation, reduce vehicle override, and limit the peak forces transmitted to the vehicle structure [6,7]. In this context, foam-filled tubular absorbers may be considered as a possible supplementary energy-absorbing concept, since they combine the progressive deformation of the metallic tube with compressive energy dissipation in the cellular foam core. The energy absorber that is proposed can be found in Figure 1. This configuration sees a foam-filled steel tube pass through a narrowing cone bushing which will cause the circular tube of PU foam to compress and the absorber convert kinetic energy into potential energy and heat through plastic deformation and friction [8].

In order to be able to model such an energy absorber, the material behavior of the PU foam must first be characterized by a hyperelastic model that is in accordance with the experimental data. With an appropriately configured material model, the absorber response can be numerically evaluated under quasi-static loading conditions as a preliminary step toward assessing its potential for railway vehicle kinetic energy absorption.

A number of studies have looked at PU foam for its energy absorption capability. Kim et al. [9] noted that rigid PU foam has characteristics of both soft foam and rubber, meaning it cannot be modelled accurately using the majority of hyperelastic models that have been developed to model incompressible materials, those with a Poisson’s ratio of 0.5. Hyperfoam models take into account the compressibility of foam and are, therefore, more suitable for this analysis. Yan et al. [10] compared the energy absorption of a foam-filled structure to an empty structure, and concluded that the energy absorption of the foam-filled tube structure is better than the empty tube. Onsalung et al. [11] followed on from this and looked at the impact response of a number of different densities of PU foam and found that energy absorption can be enhanced by using higher-density foams. A study conducted by Petre et al. [12] indicated that the Ogden foam model would be the best model to use to recreate the material behavior in Ansys [13]. The curve-fitting function in Ansys allows for the fitting of data for uniaxial, biaxial, shear, and volumetric tests. Çalışkan et al. [14] applied these test results to establish a PU material model and found that there was a significant deviation between the calculated curves and experimental results.

A prior study from Tanasković et al. [15] theorized and modelled a conical bushing collision absorber to find that the energy absorption of a foam-filled steel tube was 30% greater than that of an empty tube. One aim of this study is to verify this by reproducing these results and refine the material model. The main contribution of this study is the experimental and numerical evaluation of rigid polyurethane foam as a filler material in a tubular railway vehicle collision absorber. The work focuses on a configuration where a foam-filled steel tube is compressed through a conical bushing, rather than treating foam-filled tubes solely as general crashworthy structures. Compression tests were performed on foam samples taken from different positions within the same cylindrical sample, allowing observation of the variations in the material response across the section. The experimental data were then used to calibrate an Ogden foam numerical model in Ansys, which was applied to compare three absorber configurations: an empty steel tube, a fully foam-filled steel tube, and a foam-filled tube with a concentric steel core. Thus, the study examines both the influence of rigid PU foam on the absorber response and the suitability of the developed material model for quasi-static absorber simulations.

### 1.1. Hyperelastic Models

**Figure 2:**
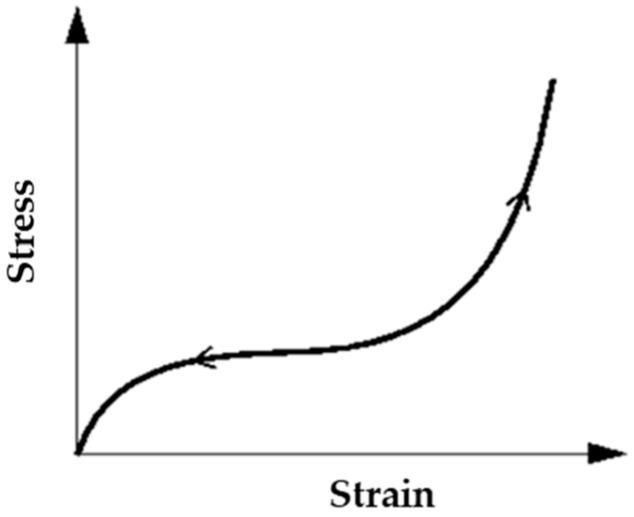
Schematic of a typical stress–strain graph for a hyperelastic material.

A hyperelastic model is used to model materials that respond elastically when subject to either compressive or tensile strains but the non-linear response of materials cannot be characterized by simple elastic moduli [16]. Figure 2 illustrates this non-linearity. Hyperelastic material models are commonly used to represent large deformations of materials with finite element analysis (FEA), and such models can be used to model the material behavior of rubbers and elastomeric foams.

Unlike for linearly elastic materials being described through material property constants such as Young’s modulus or a non-dynamic Poisson’s ratio, hyperelastic materials are defined through a strain energy density function. A strain energy density function can be used to derive the material properties and a non-linear constitutive model wherein stresses are a function of large strain deformation. The strain energy density function can be a direct function of the strain invariants or a function of the principal stretch ratios [17,18], respectively:

(1) $$\begin{array}{ccc} W=f\left(I_{1},\;I_{2},{\;I}_{3}\right) & \mathrm{or} & W=f(\lambda_{1},\;\lambda_{2},\;\lambda_{3}) \end{array}$$

where *W* is the strain energy density and *I*_1_, *I*_2_, and *I*_3_ are the three invariants of the Green deformation tensor, themselves given by the following:

(2) $$I_{1}=\lambda_{1}^{2}+\lambda_{2}^{2}+\lambda_{3}^{2}\;I_{2}=\lambda_{1}^{2}\lambda_{2}^{2}+\lambda_{2}^{2}\lambda_{3}^{2}+\lambda_{3}^{2}\lambda_{1}^{2}\;I_{3}=\lambda_{1}^{2}\lambda_{2}^{2}\lambda_{3}^{2}$$

where *λ*_i_ are the principal stretch ratios due to tensile/compressive strain. The strain energy function can be expressed as a sum of deviatoric terms and volumetric terms and defined in terms of stretch ratios as follows:

(3) $$W=f_{D}\left({\bar{\lambda }}_{1}^{\alpha },{\bar{\lambda }}_{2}^{\alpha },{\bar{\lambda }}_{3}^{\alpha }\right)+f_{V}\left(\;J\right)$$

where *α* is a material constant and *J* = λ_1_ λ_2_ λ_3_ is the volume ratio, and the deviatoric principal stretches are defined as follows:

(4) $${\bar{\lambda }}_{i}=J^{-1/3}\lambda_{i}$$

There are a number of different hyperelastic models, some based on the principal stretch ratios and some on the strain invariants. The Ogden foam strain energy function used in this study adopts the stretch ratios.

### 1.2. Ogden Foam Model

The Ogden foam model, also known as the hyperfoam model, uses a modified Ogden [19] form of the strain energy potential function for compressible foams. The function is given by [20]:

(5) $$W=\sum_{n=1}^{N}\frac{\mu_{n}}{\alpha_{n}}\left(J_{\mathrm{el}}^{\alpha_{n}/3}\left({\bar{\lambda }}_{1}^{\alpha_{n}}+{\bar{\lambda }}_{2}^{\alpha_{n}}+{\bar{\lambda }}_{3}^{\alpha_{n}}\right)-3\right)+\sum_{n=1}^{N}\frac{\mu_{n}}{\alpha_{n}\beta_{n}}\left(J_{\mathrm{el}}^{-\alpha_{n}\beta_{n}}-1\right)$$

where:

- *μ*_n_, *α*_n_ are material constants;
- *β*_n_ is the compressibility parameter;
- *J*_el_ = *J* is the determinant of the elastic deformation gradient;
- *N* is the order of fitting.

The initial shear modulus *μ*_0_ is defined by the following:

(6) $$2\mu_{0}=\sum_{n=1}^{N}\mu_{n}\alpha_{n}$$

and the initial bulk modulus *K*_0_ is defined by the following:

(7) $$K_{0}=\sum_{n=1}^{N}\mu_{n}\alpha_{n}\left(\frac{1}{3}+\beta_{n}\right)$$

In each term of the strain energy function, *β*_n_ determines the compressibility of the material. *β*_n_ is related to Poisson’s ratio *ν*_n_ by the following:

(8) $$\beta_{n}=\frac{\nu_{n}}{1-2\nu_{n}}$$

Poisson’s ratio can be obtained by measuring the lateral strain during testing.

### 1.3. Dynamic Poisson’s Ratio

Poisson’s ratio is the ratio of the transverse strain to longitudinal strain, where longitudinal strain is in the direction of the applied force (compressive deformation is considered to be negative):

(9) $$\nu =-\frac{\Delta \varepsilon_{\mathrm{trans}}}{\Delta \varepsilon_{\mathrm{axial}}}$$

The ratio is a constant for linear elastic materials. At small strains, foams may be treated as compressible solids; however, as the response of the hyperelastic foam is highly non-linear at large deformation and hardens significantly after densification, Poisson’s ratio cannot be treated as a constant; i.e., Poisson’s ratio will vary with the strain.

The dynamic Poisson’s ratio is of high interest in this study as it is to be used in order to find values for the compressibility parameters that form part of the hyperelastic strain energy density function. A study from Sanborn et al. [21] has shown how the Poisson’s ratio of a hyperelastic foam material was relatively constant around 0.2, but, when subject to large deformation into material densification, the ratio changed significantly up to 0.45 and near incompressibility.

## 2. Experimental Procedure

### 2.1. Material

Polyurethanes are a class of polymer with a wide range of uses and exist in a variety of forms including flexible foams, rigid foams, and elastomers depending on the molecular structure. PU foam is created by reacting a liquid isocyanate with two or more isocyanate groups per molecule with liquid polyol, an organic compound containing multiple hydroxyl groups per molecule in the presence of a catalyst [22]. It is the influence of the type of isocyanates and polyols that give forms of polyurethane a wide range of properties. PU foams are made up of long chains, contributed by the polyol, with intermediate crosslinking.

In this research, the PU foam was produced in circular tubes of diameter 85 mm and length 200 mm, with a density of 175 kg/m^3^, at the Laboratory of Polymers at the Faculty of Technology and Metallurgy, University of Belgrade.

### 2.2. Material Cutting

**Figure 3:**
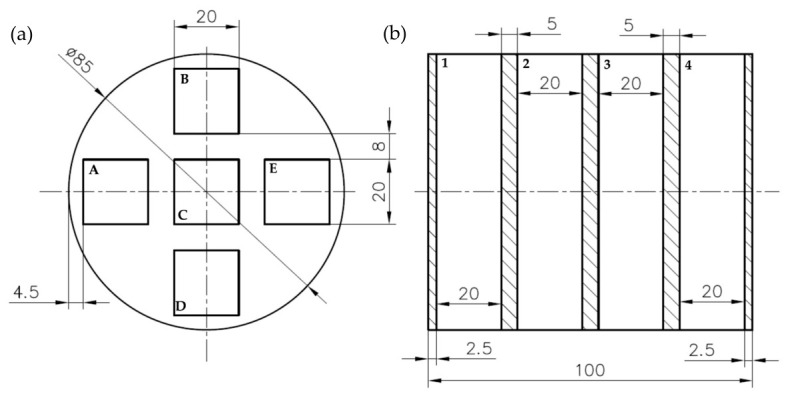
(**a**) Cutting pattern from cylindrical sample. (**b**) Cubic samples cut from one 100 mm column.

A 100 mm length of the foam cylinder was initially cut into twenty cubic 20 mm samples. Figure 3 shows how the cubic samples were cut from the sample.

This configuration was chosen to test whether cubic samples taken from the center (C) exhibit different material properties to samples taken from other locations in the cylinder (A, B, D, E). As seen in the figure, each sample is given a letter and a number corresponding to where in the cylinder it has come from. Cubic samples indicated with a ‘1’ were closest to the circular surface of the cylinder. It was important that each cubic sample was tested in the same orientation since foams are usually anisotropic. To ensure this, the cubic samples were all labelled on the same faces with the same orientation.

### 2.3. Material Testing

**Figure 4:**
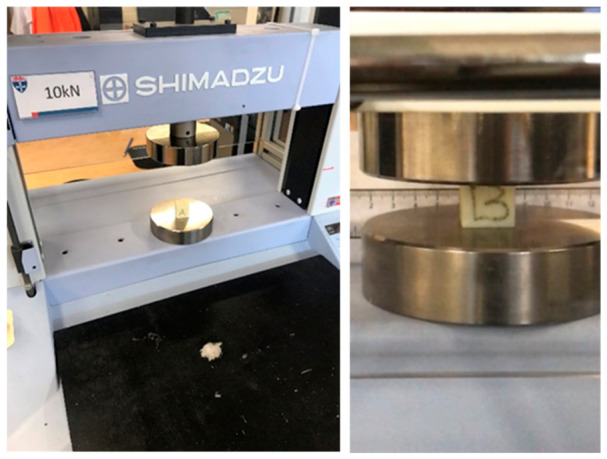
Mechanical testing using Shimadzu 10 kN load cell.

All material testing was conducted in accordance with EN ISO 3386-2:1998/A1:2010 [23]. Uniaxial compression tests were performed on cubic samples using a Shimadzu tensile test frame with a 10 kN load cell (Figure 4). Tests were conducted at room temperature with a constant crosshead speed of 25 mm/min until the predefined stroke was reached. Samples labeled as type 1 were compressed to 2 mm, type 2 to 4 mm, type 3 to 8 mm, and type 4 to 12 mm, corresponding to approximately 60% strain. A preload of about 1 N was applied before each test, and force, stress, strain, and stroke data were recorded at 0.01 s intervals.

To evaluate transverse strain, each test was filmed with a slow-motion camera (240 fps). A ruler affixed to the rear screen enabled measurement of lateral extension, allowing assessment of Poisson’s ratio variation with strain. These results were then used to determine the compressibility parameters of the Ogden foam model [24].

## 3. Experimental Results

### 3.1. Stress–Strain Curves

**Figure 5:**
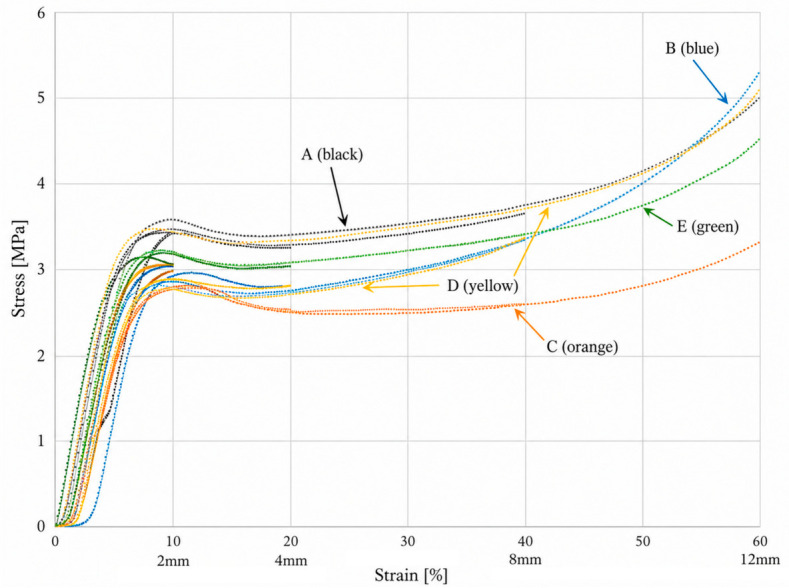
Compressive stress–strain curves for all cubic samples.

Figure 5 shows the compression curves obtained from testing, with each graph containing four curves labeled A to E. Overall, the curves exhibit a similar trend.

**Figure 6:**
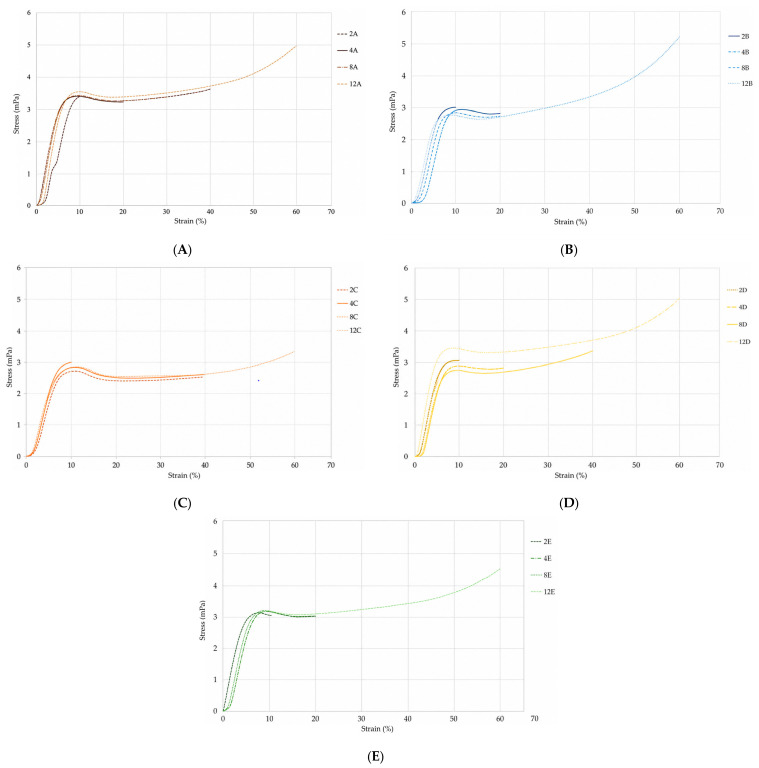
Compressive stress–strain curves for samples labelled from (**A**–**E**).

Although the curves generally follow the same shape, sample C deviates noticeably from the others. Up to strains of approximately 8%, all specimens display a region of linear elasticity; however, these linear regions do not intersect at the origin, likely due to the relatively small preload applied. To address this when determining the material constants for the Ogden foam model, arbitrary offsets will be introduced. The similarity among curves A, B, D, and E indicates consistent and reliable results, as shown in Figure 6.

For specimens tested to a 12 mm stroke, excluding sample C, the data range can be quantified. The maximum variation occurs at a strain of about 10%, where samples A and B differ by 0.7 MPa. An examination of curve D reveals a wider spread between samples compared to the other cases. The specimen tested to a 12 mm stroke shows a maximum linear elastic stress 0.71 MPa higher than the other specimens from the same group, whereas the differences for cases A, B, and E are 0.1 MPa, 0.19 MPa, and 0.09 MPa, respectively.

### 3.2. Consolidation of C Sample Results

Specimens taken from the center of the circular tube exhibited lower stress than the other cases once strains exceeded 20%. In the 4 mm stroke tests, the stress response of sample C was approximately 14% lower than the average of the other specimens. At the 8 mm stroke, the difference increased to 24%, and, at the 12 mm stroke, the stress was 33% lower, as summarized in Table 1.

The reduced stress response of the C samples became more pronounced at higher strains. This behavior may be associated with the spatial heterogeneity that develops during foam expansion and curing, including local density variations and differences in the cell morphology between the central and peripheral regions of the cylinder. Since the local density and microstructure were not measured separately for each specimen location, this explanation should be interpreted as a possible mechanism rather than a confirmed cause.

The A, B, D, and E samples were used for the primary material calibration because they represented the majority of tested locations and showed a more consistent mechanical response than the central C samples. However, this selection is a modelling assumption. The real foam cylinder contains spatial heterogeneity, and, therefore, a single homogeneous material model cannot fully represent all local foam regions.

Accounting for such spatial variation in material properties within a single foam structure is challenging and complicates implementation in a constitutive model. One practical approach, adopted in the present study, was to exclude the data from cubic samples labeled C while acknowledging that the resulting material model cannot fully capture the behavior of the complete circular foam section. Alternatively, future models could represent the central region as a reduced-stiffness zone or introduce spatially varying foam properties, but this would require additional density and microstructural data from different specimen locations.

### 3.3. Curve Regions

**Figure 7:**
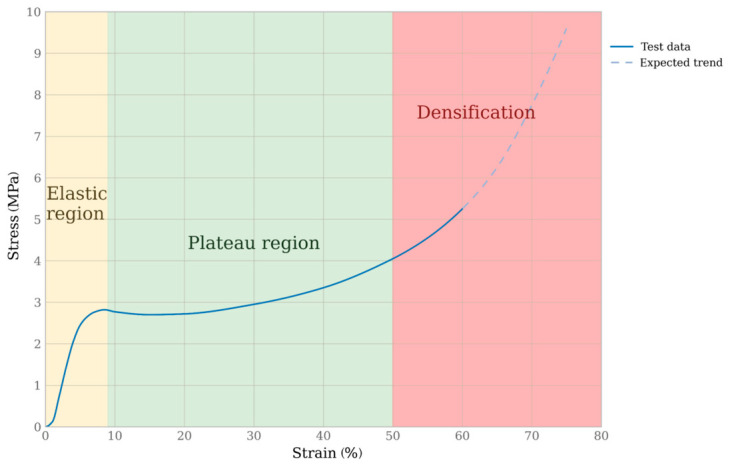
Stress–strain curves of cubic sample B.

Each stress–strain curve for those samples tested to a 60% strain shows the predicted conventional hyperelastic curve with three distinct regions: a linear region, a plateau, and a densification region (Figure 7). A representative curve has been extrapolated to illustrate the expected behavior based on cubic sample B.

Different mechanisms of deformation occur between these regions. The elastic region shows a region of Hookean (linear elasticity) behavior which is controlled by atomic cell wall bending [2]. As a linear relationship, this region can be characterized by moduli such as the Young’s modulus of the material, and the initial slope of the PU foam response. As previously mentioned, the PU foam is anisotropic and may not have the same properties in each plane. The plateau region is initiated by the elastic collapse of the cells causing a stress relaxation—this can be seen in Figure 6 as the stress decreases slightly at a strain of near 10%. During the plateau region, cells continue to collapse right up to the densification region where opposing cell walls are compressed together and further strain causes an increased rate of increasing stress.

## 4. Numerical Model and Simulations

The aim of the finite element analysis (FEA) was first to replicate the uniaxial compression tests in order to see how accurately the Ogden foam model can replicate the force vs. stroke data of the cubic sample tests. After a suitable material model is created, the collision absorber can be modelled to analyze its energy absorption capabilities.

All FEA work was done using the Ansys Mechanical 2019 R3 FEA software package [14]. An Ogden foam third-order model was selected to model the behavior of the PU foam.

### 4.1. Accounting for Irreversible Compression

The schematic graph in Figure 2 suggests that stresses and strains in foam materials under loading are completely reversible, implying that loading and unloading curves would coincide during cyclic loading. This assumption also implies incompressibility, consistent with the approach of many hyperelastic models, which often treat materials as incompressible or nearly incompressible. However, modelling foam compression remains challenging, especially when implementing non-standard strain energy equations in Ansys.

**Figure 8:**
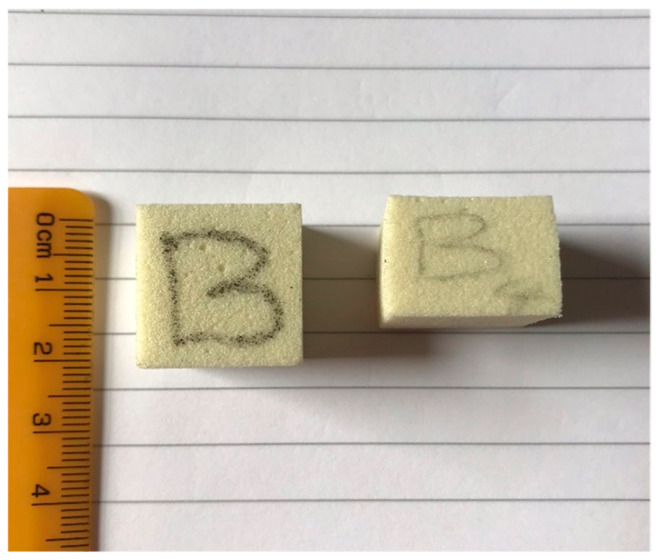
Cubic samples B1 and B4 post-testing.

The experimental inspection of the foam cubic samples after testing indicates that compression is not fully reversible. Figure 8 compares a specimen tested to a 10% strain with one tested to a 60% strain, both examined shortly after testing, and shows clear evidence of irreversible deformation.

In the case of fully reversible compression, the cubic samples would return to their original shape. Instead, they show irreversible deformation. While this does not present a problem for the intended application as a collision absorber, since the component is designed for single use during a large impact, it complicates the development of a fully accurate material model to represent the foam behavior across the entire stress–strain curve. Once the foam begins to collapse, it no longer follows the same hyperelastic path. With this in mind, a model can be constructed to best capture the strain levels relevant to the collision absorber, providing the most accurate representation of the force response within its functional range.

Table 2 presents the dimensions and the calculated Poisson’s ratio of the 20 mm cubic samples during and after testing. The Poisson’s ratio increases as the stroke increases, as expected. As more compression occurs, the Poisson’s ratio gets closer to 0.5 and incompressibility. Evidence of irreversible compression at the 8 mm and 12 mm stroke provides validity to previous statements regarding the foam’s structural collapse. At the 4 mm stroke, the shape of the cubic samples is very similar to their original dimensions, indicating most of the deformation was within the elastic limit of the foam. Data for cubic samples tested to 2 mm are not included in the table due to no differences in dimensions being quantifiable. For sample C4, the axial width post-test is considerably larger than other samples tested to the same stroke.

### 4.2. Recreating Uniaxial Test

The values for stress and strain from testing are values for the engineering stress and strain. In order to input these correctly into Ansys, they must be adjusted to represent the true stress and true strain [25]. The equations below allow for this adjustment.

(10) $$\sigma_{\mathrm{true}}=\sigma_{\mathrm{engineering}}\times \left(1+\varepsilon_{\mathrm{engineering}}\right)\;\varepsilon_{\mathrm{true}}=\ln\left(1+\varepsilon_{\mathrm{engineering}}\right)$$

The average stress–strain data used for material calibration were obtained from the A, B, D, and E specimen groups, while the C samples were excluded from the primary calibration dataset. This decision was made because the C samples, taken from the central region of the cylindrical foam sample, consistently showed a lower stress response than those from the surrounding regions. As discussed above, this difference became more pronounced with increasing compression, indicating that the central region did not represent the dominant response observed across the tested specimen locations.

The exclusion of the C samples was therefore treated as a modelling assumption rather than a correction to improve the numerical fit. The non-central samples were used because they provided a more representative average response for the majority of tested specimen locations and because the absorber model was intended to describe the general response of the foam-filled tube. This assumption introduces a limitation, since the real foam tube contains material non-uniformity across its section. Future work should therefore include either a spatially varying foam material model, or a sensitivity analysis comparing absorber simulations calibrated with and without the central C specimen data.

**Figure 9:**
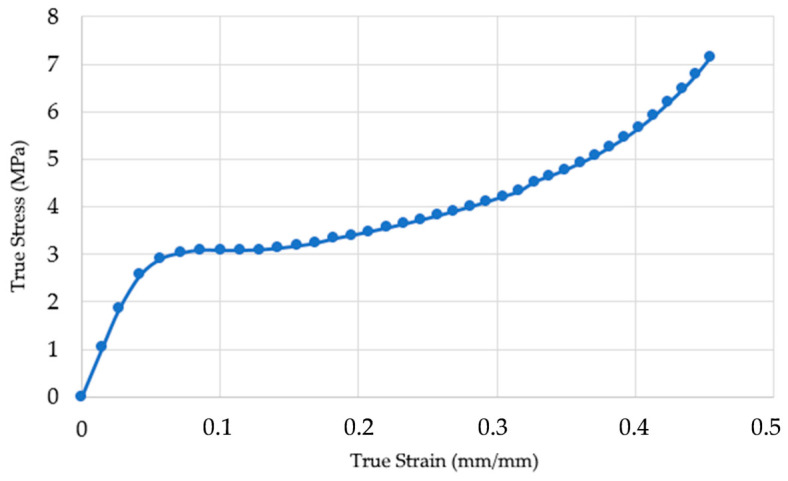
True stress vs. strain curve.

This was then used as the uniaxial test data in Ansys when defining the hyperelastic properties of the PU foam material, and a graph of the average data is shown in Figure 9.

The constants in Equation (10) were found using the results of the experimental investigation. The compressibility parameters *β*_n_ were found using Equation (8). The material constants were refined through iterative means:

(11) $$\mu_{1}=4.4\times 10^{6}\;\alpha_{1}=20\;\beta_{1}=0.452\;\mu_{2}=4.4\times 10^{6}\;\alpha_{2}=-10\;\beta_{2}=0.542\;\mu_{3}=9.9\times 10^{6}\;\alpha_{3}=0.45\;\beta_{3}=1.082$$

**Figure 10:**
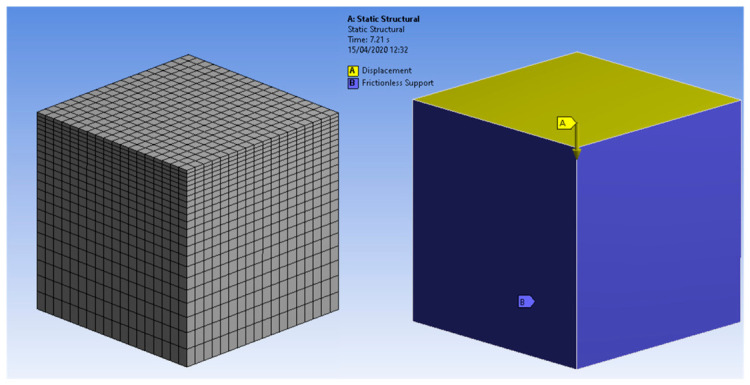
Boundary conditions and mesh for eighth cubic sample.

An eighth of the cubic sample was modelled with three frictionless supports and a displacement boundary condition to compress the cubic sample, with the large stroke setting on. The boundary conditions and mesh are presented in Figure 10. The meshed cube has 22 elements, bias factor 5, along the edges parallel to the load direction and 18 elements along the perpendicular edges. The total node number is 31,977. During tests, the reaction force was measured on the defined stroke.

**Figure 11:**
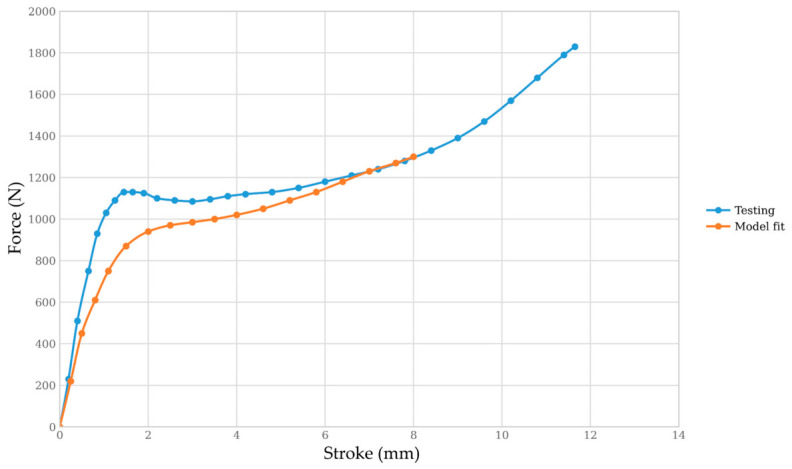
Force vs. stroke curves for PU foam cubic sample.

Figure 11 shows the force vs. stroke data from testing and the third-order Ogden foam model fit.

The model follows a similar trend to the testing data. However, it is not able to represent the peak force at the linear elastic region and the following force relaxation. A stroke above 8 mm is not considered as these strains will not occur in the application of the collision absorber. The average root mean square (RMS) error can be calculated as per Equation (12) [26] in order to compare the experimental force and the FE predicted force. The RMS error over *M* test measurements up to 8 mm is found to be 8.5%:

(12) $$\mathrm{RMS}\;\mathrm{Error}=\frac{1}{M}\sqrt{\sum_{m=1}^{M}\frac{F_{m\left(\mathrm{Measured}\right)}-F_{m(\mathrm{FE})}}{F_{m\left(\mathrm{Measured}\right)}}}$$

The model is not as accurate in representing the forces at small strokes. The RMS error of the model up to a 3 mm stroke is 19.1%, compared to 6.3% error from 3.5 mm to 8 mm.

The compressibility parameters do have an effect on the force reaction of the stroke boundary, but they also control the amount by which the material protrudes transversely under loading. As mentioned previously, these parameters were calculated using Poisson’s ratio where the values were estimated from videos of the testing. In order to assess the validity of the compressibility parameters, simple percentage calculations can be made to compare the transverse deformation of a cubic sample during testing and in the model. It is expected that they should be accurate to the values taken from testing. Table 3 illustrates this for the stroke in one positive direction only.

An inspection of these values show that the model is accurate in terms of modelling transverse deformation as expected. This does not mean the model is an accurate representation of the true case, however, as there is human and measurement error to be considered. The apparatus used to measure this during testing was a phone camera and a ruler due to the limitations at the time of testing.

**Figure 12:**
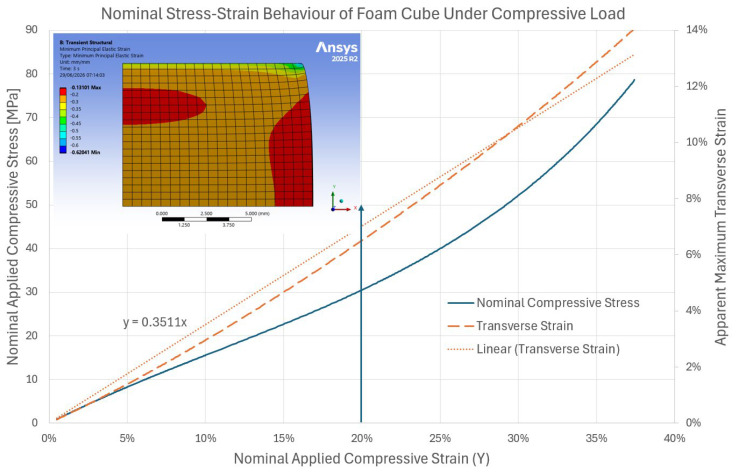
Nominal stress–strain behavior derived from ANSYS (ANSYS Workbench Mechanical, Release 2019 R3 and Release 2025 R2) Transient Structural model of 10 mm cube of material Ogden Foam First-Order Foam (μ_1_ = 14 MPa, α_1_ = 10, β_1_ = 0.5) with three frictionless constraints and a displacement control. Element size is 0.5 mm and the final simulation reached 37% compressive strain. Inset: Minimum principal strain (external/free cube face) at 20% compression.

Transverse protrusion, i.e., ‘barreling’, occurs also with the first-order model used in Section 4. The nominal stress–strain behavior derived from the compression of a foam cube is shown in Figure 12.

The cubic sample during testing can be seen to ‘barrel’ but predominantly in the upper half whilst the bottom half of the cubic sample seems to compress more and deform to a lesser extent. This asymmetry may be caused by the experimental setup or by the material property variation within the samples, and merits careful observation in future tests.

The FE simulation of the PU foam cubic sample has given a reasonable material model that can be used in the collision absorber in order to analyze the foam’s ability to absorb energy. The model provides an acceptable approximation of the experimental response within the strain range relevant to the absorber simulation, although allowed deviations persist at small strokes and in representing local deformation.

The sensitivity of the finite element model to element size is shown in Table 4. As the element size reduces from 1.0 mm to 0.5 mm, the results for transverse displacement and force reaction tend asymptotically to ‘correct’ values. The error is less than 0.2%. However, the model can quickly become unstable near sharp stress gradients.

### 4.3. Model for Collision Absorber

**Figure 13:**
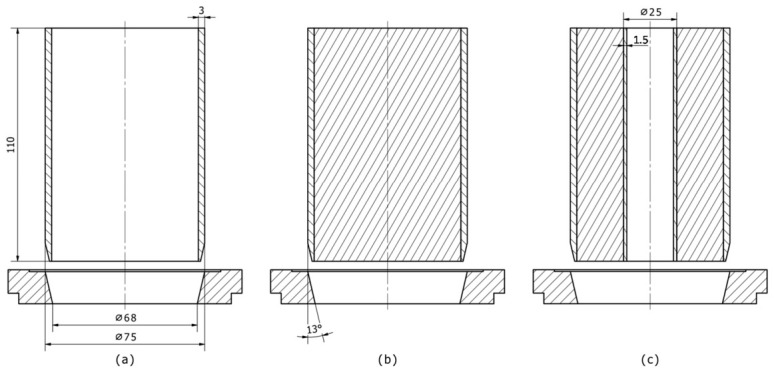
Section view of (**a**) empty tube absorber, (**b**) foam-filled absorber, and (**c**) concentric core absorber (all units in mm).

With a suitable material model for PU foam, this can now be implemented to a model for the collision absorber. To follow a similar previous study, the collision absorber is to be modelled as three alternatives: one as an empty metal tube, one as a PU foam-filled tube, and one as a PU foam-filled tube with its core removed and replaced with a smaller diameter metal tube. Drawings and dimensions of the collision absorbers to be analyzed can be found in Figure 13.

The foam is enclosed by a steel tube with a thickness of 3 mm. In the absorber with the foam core removed, a steel tube with a thickness of 1.5 mm is present. The steel tubes were given a yield strength of 315 MPa and the steel bushing a yield strength of 430 MPa.

**Figure 14:**
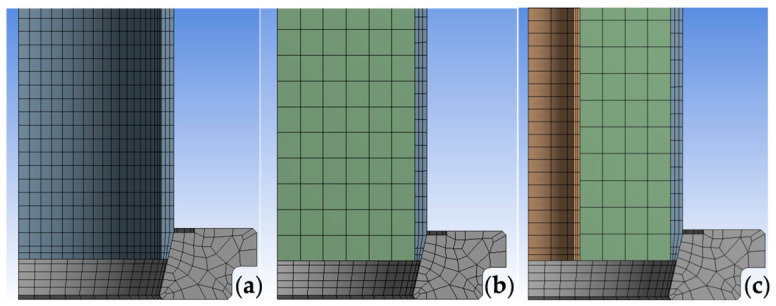
Collision absorber meshes: (**a**) empty tube, (**b**) foam-filled tube, and (**c**) concentric filled tube.

In order to simplify the model, a quarter of the collision absorber assembly was modelled using frictionless supports. A contact region between the steel tube and the bushing was created and defined as frictional, with a friction coefficient of 0.15 with the behavior selected as asymmetric. The contact was selected to be solved through augmented Lagrangian methods. Careful consideration was made when defining the mesh. Preliminary work indicated that a too-fine mesh caused the model to not converge—this was found to be due to nodal overlap during compression. Mesh definitions used in each model is presented in Figure 14.

As with earlier FEA work, large strokes are turned on. The bushing is held using a fixed support and a stroke boundary condition applied on the top of the tube through the bushing.

### 4.4. Strain in the Collision Absorber

**Figure 15:**
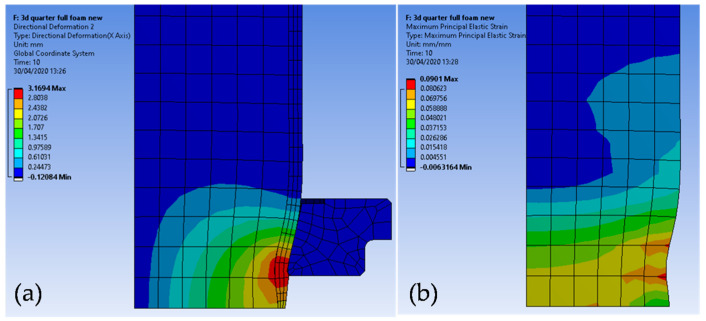
(**a**) Directional deformation of foam-filled collision absorber. (**b**) Maximum principal strain in foam.

Figure 15a,b show the directional deformation and the maximum principal strain (around 9%) in the foam-filled absorber. This indicates that the previous material model for the foam is not entirely suitable for this study, because, as previously mentioned, the material model that was selected is not wholly representative of the experimental data at strains up to 11%.

Due to this, it is no longer necessary to model the foam using a third-order function and can instead be replaced by a first-order function because any differences in the predicted load between two accurate functions will be minimal at strains of up to 11% so a higher order is unnecessary. The third-order function allows for the complex response of the foam to be modelled across larger strains which is no longer necessary for the study. This raises the idea of implementing the orthotropic material behavior observed into future models of the collision absorber depending on the amount of strain that is predicted by the FEA. This can be done by fitting the predicted load data to the experimental data up to the maximum strain predicted.

**Figure 16:**
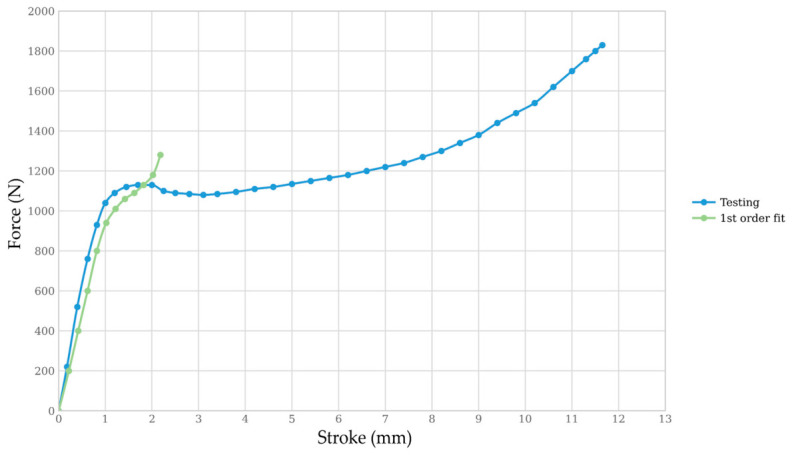
First-order model fit for strains of up to 11% in cubic sample.

Figure 16 shows the curve fit of the first-order model to be accurate up to a stroke of 2 mm on a 20 mm cubic sample, equivalent to a 10% strain.

For the Ogden foam first-order material model used in Figure 16, the constants used in the material model for the foam in the collision absorber are as follows:

(13) $$\begin{array}{ccc} \mu_{1}=14\times 10^{6} & \alpha_{1}=10 & \beta_{1}=0.5 \end{array}$$

### 4.5. Energy Absorption

**Figure 17:**
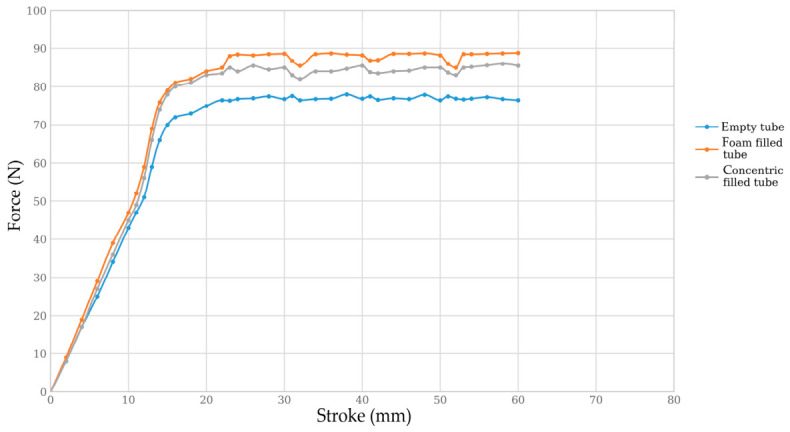
Force vs. stroke curve from numerical simulation of three collision absorbers.

Figure 17 is a graph of the force vs. stroke for the three different energy absorber models up to a stroke of 60 mm.

A steady force response with small deviations occurs at around 25 mm. This steady force for the empty tube is roughly 77 kN, and, for the case of the foam-filled tube and the concentric filled tube, the forces are roughly 89 kN and 85 kN, respectively. A greater force is required in the foam-filled absorbers due to higher compression forces being required to overcome the elastic and plastic deformation of the steel tube as the foam provides a constraint mechanism on the tube. To assess the three collision absorbers for their energy absorption capabilities, the energy absorbed can be calculated as follows:

(14) W=∑F(h)δh

where:

- *W* = the absorbed energy;
- *F*(*h*) = the force response at a defined stroke;
- *δh* = the increment of a stroke.

Table 5 contains key parameters for the evaluation of energy absorption between the three absorbers up to a stroke of 60 mm. The *F*_avg_ column takes the average force response from a 25 mm to 60 mm stroke.

As can be seen from the values in the table and an inspection of Figure 16, the trend agrees with the findings of previous studies [10,11] that the presence of high-density foams increases the energy absorption capabilities of a collision absorber. In this study, the two foam-filled absorbers have a greater energy absorption power than that of an empty tube, with the full foam tube absorber being able to absorb ≈16% more energy than the empty tube, whilst the concentric filled absorber can absorb around 9.5% more than the empty tube. Among the three simulated configurations, the fully foam-filled absorber exhibited the highest quasi-static energy absorption. However, its suitability for railway collision applications requires additional dynamic testing and the validation of the complete absorber assembly.

Although the numerical results indicate that foam-filled configurations absorb more energy than the empty tube, the absorber simulations should be interpreted as comparative numerical predictions based on a calibrated foam material model, not as fully validated absorber-level results. The complete absorber assembly was not experimentally tested in this study. The predicted absorbed energies may be affected by uncertainty in the friction coefficient, contact stiffness, mandrel and fixture compliance, steel material hardening behavior, foam heterogeneity, geometric imperfections, mesh resolution, and boundary-condition simplifications. Full absorber-level force–stroke testing is therefore required to validate the numerical predictions.

## 5. Conclusions

This study investigated the energy absorption behavior of rigid polyurethane foam used as a filler material in a tubular railway vehicle collision absorber. Compression tests on 20 mm cubic samples showed that the PU foam exhibited the expected non-linear response of a cellular material, with an initial elastic region, followed by a plateau and progressive densification. The test results also showed that samples taken from the central region of the cylindrical foam sample had a lower stress response than those taken from the outer regions, indicating that the foam properties were not fully uniform across the section.

The experimental compression data were used to calibrate an Ogden foam material model for finite element analysis. Although the higher-order Ogden model reproduced the general force–stroke trend of the compression tests, deviations were observed at small strokes and in the representation of local deformation. Since the maximum principal strain predicted in the foam-filled absorber was approximately 11%, a first-order Ogden foam model calibrated over the corresponding low-strain range was selected for the final absorber simulations.

Numerical simulations were performed for three absorber configurations: an empty steel tube, a fully foam-filled steel tube, and a foam-filled tube with a concentric steel core. The fully foam-filled configuration showed the highest energy absorption, absorbing 4.61 kJ over a 60 mm stroke. The empty tube absorbed 3.96 kJ, while the concentric-core configuration absorbed 4.38 kJ. Therefore, the fully foam-filled tube increased the absorbed energy by approximately ≈ 16% compared with the empty tube, while the concentric-core configuration produced an increase of approximately 9.5%.

The results show that rigid PU foam can improve the quasi-static energy absorption capacity of a tubular railway collision absorber by increasing the deformation resistance of the tube. However, these findings are based on quasi-static material testing and a numerical simulation of the absorber. Further work should include dynamic testing of the foam, an improved measurement of the transverse strain, mesh and contact sensitivity studies, and experimental validation of the complete absorber assembly under loading conditions representative of the railway collision eve. Finally, future studies should investigate the dynamic effects of the foam density and absorber geometry. Additionally, the tube length, available installation space, and tube diameter-to-thickness ratio should be considered when developing an absorber with a suitable force response and energy absorption capacity for railway vehicle applications.

## Figures and Tables

**Table 1:** Stress data comparison between cubic samples.

Stroke	Stress at Max Stroke (MPa)						C to Average (%)
	A	B	C	D	E	Average ABDE	
**2 mm**	3.42	3.04	3.00	3.05	3.05	3.14	4.70
**4 mm**	3.25	2.84	2.56	2.83	3.04	2.99	14.36
**8 mm**	3.65	3.35	2.62	3.37	3.41	3.44	23.92
**12 mm**	4.98	5.24	3.31	5.06	4.51	4.95	32.99

**Table 2:** 20 mm cubic sample testing dimensions.

Sample	Stroke (mm)	Max Width During Testing (mm)	Poisson’s Ratio	Transverse Width Post Testing (mm)	Axial Width Post Testing (mm)
**A2**	4	-	-	20	19
**B2**		20.8	0.2	20	19.4
**C2**		21	0.25	20.2	19.2
**D2**		21	0.25	20	19.5
**E2**		21	0.25	20.1	19
**AVG2**		**20.95**	**0.2375**	**20.06**	**19.22**
**A3**	8	22	0.25	21	17.2
**B3**		22	0.25	20.2	17.5
**C3**		21.8	0.225	20.5	17.9
**D3**		22.2	0.275	20.6	17.6
**E3**		22.4	0.3	21	17.5
**AVG3**		**22.08**	**0.26**	**20.66**	**17.54**
**A4**	12	24	0.333	22	14.8
**B4**		24.2	0.35	21.2	15
**C4**		24.2	0.35	21	16
**D4**		24	0.333	21.8	14.8
**E4**		-	-	21.5	14.9
**AVG4**		**24.1**	**0.341667**	**21.5**	**15.1**

**Table 3:** Directional deformation transverse to load (values rounded to 2 d.p.).

Stroke	Avg. from Tests	Model	Error
**2 mm**	0.25 mm	0.25 mm	0.48%
**4 mm**	0.48 mm	0.53 mm	9.68%
**8 mm**	1.04 mm	1.00 mm	3.85%
**12 mm**	2.05 mm	1.98 mm	3.42%

**Table 4:** Sensitivity to element size in ANSYS Transient Structural. A 10 mm cube of material Ogden Foam First-Order Foam (μ_1_ = 14 MPa, α_1_ = 10, β_1_ = 0.5) with three frictionless constraints and a displacement control; force reaction and maximum transverse displacement recorded at 2 mm compression for comparison. The final simulation reached 37% compressive strain.

Element Size	Applied Y Disp.	Max X Disp.	Y Force Reaction
mm	mm	mm	kN
1.0	−2.0	0.65191	−3.0559
10/13 ≈ 0.77	−2.0	0.65163	−3.054
10/17 ≈ 0.59	−2.0	0.65142	−3.0526
0.5	−2.0	0.65133	−3.0519
	−3.7387	1.404	−7.8395

**Table 5:** Force and energy absorption parameters for simulated absorbers.

Absorber Type	F_max_ (kN)	F_avg, steady response_(kN)	W (kJ)
Empty tube	78.51	77.37	3.96
Full foam tube	88.93	88.17	4.61
Concentric tube	86.44	84.61	4.38

## Data Availability

The original contributions presented in this study are included in the article. Further inquiries can be directed to the corresponding author.
